# Measures of insulin sensitivity, leptin, and adiponectin concentrations in cats in diabetic remission compared to healthy control cats

**DOI:** 10.3389/fvets.2022.905929

**Published:** 2022-07-29

**Authors:** Susan Gottlieb, Jacquie S. Rand, Katsumi Ishioka, Daniel A. Dias, Berin A. Boughton, Ute Roessner, Ziad Ramadan, Stephen T. Anderson

**Affiliations:** ^1^The Cat Clinic, Brisbane, QLD, Australia; ^2^School of Veterinary Science, The University of Queensland, Gatton, QLD, Australia; ^3^Australian Pet Welfare Foundation, Kenmore, QLD, Australia; ^4^School of Veterinary Nursing and Technology, Faculty of Veterinary Science, Nippon Veterinary and Life Science University, Tokyo, Japan; ^5^Discipline of Laboratory Medicine, School of Health and Biomedical Sciences, Royal Melbourne Institute of Technology (RMIT) University, Bundoora, VIC, Australia; ^6^School of BioSciences, The University of Melbourne, Melbourne, VIC, Australia; ^7^Australian National Phenome Centre, Murdoch University, Murdoch, WA, Australia; ^8^Nestlé Purina Research, St Louis, MO, United States; ^9^School of Biomedical Sciences, The University of Queensland, St Lucia, QLD, Australia

**Keywords:** diabetic remission, cats, insulin, leptin, adiponectin, insulin sensitivity, insulin resistance, metabolomics

## Abstract

**Objectives:**

Firstly, to compare differences in insulin, adiponectin, leptin, and measures of insulin sensitivity between diabetic cats in remission and healthy control cats, and determine whether these are predictors of diabetic relapse. Secondly, to determine if these hormones are associated with serum metabolites known to differ between groups. Thirdly, if any of the hormonal or identified metabolites are associated with measures of insulin sensitivity.

**Animals:**

Twenty cats in diabetic remission for a median of 101 days, and 21 healthy matched control cats.

**Methods:**

A casual blood glucose measured on admission to the clinic. Following a 24 h fast, a fasted blood glucose was measured, and blood sample taken for hormone (i.e., insulin, leptin, and adiponectin) and untargeted metabolomic (GC-MS and LC-MS) analysis. A simplified IVGGT (1 g glucose/kg) was performed 3 h later. Cats were monitored for diabetes relapse for at least 9 months (270 days).

**Results:**

Cats in diabetic remission had significantly higher serum glucose and insulin concentrations, and decreased insulin sensitivity as indicated by an increase in HOMA and decrease in QUICKI and Bennett indices. Leptin was significantly increased, but there was no difference in adiponectin (or body condition score). Several significant correlations were found between insulin sensitivity indices, leptin, and serum metabolites identified as significantly different between remission and control cats. No metabolites were significantly correlated with adiponectin. No predictors of relapse were identified in this study.

**Conclusion and clinical importance:**

Insulin resistance, an underlying factor in diabetic cats, persists in diabetic remission. Cats in remission should be managed to avoid further exacerbating insulin resistance.

## Introduction

Remission in diabetic cats has been previously described by our group as maintaining euglycaemia for at least 2 weeks after insulin therapy has ceased, and can be achieved in many diabetic cats. Newly diagnosed cats with type-2 like diabetes enrolled in studies with early intervention, intensive long-acting insulin therapy, and low carbohydrate diets have reported the highest remission rates. Relapse requiring further insulin therapy has been reported in ~25–30% of cats in remission. The majority (76%) of cats in remission have been previously reported to have impaired glucose tolerance, with some (19%) having impaired fasting glucose, indicating these cats do not have normal glucose metabolism or clearance (1).

Cats in diabetic remission have been demonstrated to have significant (*P* ≤ 0.05) differences in several key metabolites compared to control cats, such as an increase in glucose and decreases in many glucogenic amino acids (i.e., alanine, asparagine, aspartate, glutamine, methionine, proline, serine, tyrosine, and valine) indicating a possibly increased consumption for gluconeogenesis (2).

Newly diagnosed diabetic cats are approximately six times less sensitive to insulin than non-diabetic cats, which is similar to that reported in humans (3). Insulin resistance (decreased insulin sensitivity) is due to a reduction in the signalling and biochemical actions normally modulated by insulin (4, 5). This results in decreased uptake of glucose by skeletal muscle, the largest tissue utilising glucose in the body, and reduced inhibition of hepatic gluconeogenesis. Thus, there is decreased clearance of glucose from the blood by skeletal muscle and increased glucose production by the liver, resulting in hyperglycaemia (6). Decreased insulin receptor expression on the cell membrane in target tissues, and receptor defects, lead to mild insulin resistance (7). More severe insulin resistance occurs if post-receptor defects develop, leading to decreased glucose transport activity within the cell and increases the severity of hyperglycaemia (7). In a healthy state, increased insulin decreases hepatic gluconeogenesis by supressing gene expression for gluconeogenic enzymes. Insulin resistance reduces the ability of insulin to decrease production of these enzymes, leading to a loss of homeostasis of hepatic gluconeogenesis (8). Fasting insulin concentrations are an indicator of insulin sensitivity until pancreatic beta cell failure ensues and glucose concentration increases. However, there are no reports in the literature comparing insulin concentrations between cats in diabetic remission.

Increased leptin and decreased adiponectin concentrations have been reported in obese cats (9–12), and this inverse relationship appears to be more profound in newly diagnosed diabetic cats suggesting these adipokine hormones may be involved in the pathogenesis of diabetes in cats (13). Leptin is known to regulate energy intake through the central nervous system by decreasing food intake and increasing energy expenditure, as well as influencing fat and carbohydrate metabolism (14). Interestingly, in cats, increased plasma leptin concentrations are associated with insulin resistance, regardless of whether cats were lean or obese (15). In contrast, adiponectin regulates insulin action and energy homeostasis through increasing glucose utilisation and fatty acid oxidisation by muscle, and decreasing hepatic glucose production (14). Adiponectin concentrations are reported to be decreased in obese humans and cats, and increased intra-abdominal fat is more strongly associated with decreased concentrations compared to subcutaneous fat (6). As obesity is a risk factor for the development of diabetes in cats, adiponectin may also have a significant role in the development of this disease (16).

The aims of this study were to compare serum concentrations of insulin, leptin, and adiponectin between diabetic cats in remission and healthy control cats, as well as assessing leptin:adiponectin ratios and calculating insulin sensitivity to compare between groups. Additionally, the study aimed to assess if hormone concentrations were predictors of relapse.

## Materials and Methods

### Study design

This was a retrospective and prospective cohort study that recruited client-owned diabetic cats in remission from a feline-only veterinary hospital (“remission cats”). Practice records were reviewed from January 2005 to April 2010 to retrospectively identify previously insulin-treated diabetic cats in which insulin administration had ceased, and cats were also recruited prospectively from April 2010 to August 2012. To enrol in the study, cats must have 1. Been previously diabetic requiring insulin therapy; 2. have recorded at least one non-fasted casual glucose concentration of ≤ 6.5 mmol/L (≤ 117 mg/dL) a minimum of 2-weeks after cessation of insulin treatment; and 3. Have no clinical signs of diabetes prior to inclusion in the current study. Remission date in this study was defined as 14 days after the date administration of insulin ceased, and only cats in their first remission were included. Remission dates for enrolled cats were from April 2006 to August 2012. The cats in this study were assumed to have type-2 diabetes unless clinical signs at diagnosis indicated testing for other specific types of diabetes. Cats with other specific types of diabetes were included in this study (e.g., pancreatitis, acromegaly, corticosteroid induced). Clinically healthy owned cats presenting to a feline-only or The University of Queensland Veterinary Teaching Hospital Veterinary Hospital (“control cats”) were frequency-matched to the remission cats based on age and body condition score and enrolled in the study.

Twenty cats in remission (with a median remission time of 110 days, range of 10 days−4 years) and 32 control cats were enrolled into the study (Table 1). Both remission and control cats were admitted to hospital after enrolment. Casual blood glucose (measured anytime in relation to eating), body weight, and body condition score were all recorded (17). A 3 mL jugular sample was collected to send to an external laboratory^1^ [for haematology, serum biochemistry, serum fructosamine, total thyroxine (t4), and feline pancreatic lipase immunoreactivity], and an *in-house* urinalysis was performed. Cats were hospitalised for 24 h, during which time all food was withheld. Another 3 mL fasted jugular sample was collected for further biochemical and metabolomic analyses of serum samples. Blood was collected and placed into a red-top serum tube, centrifuged for 8 min at 1,500 g, and the separated serum placed into 1 mL Eppendorf tubes. Samples were stored at −80°C and transported on dry ice to the laboratory for analysis. An intravenous catheter was also placed in the cephalic vein at the time. Cat signalment, body condition score, fasting blood glucose, glucose tolerance measures, fructose concentrations, and feline pancreatic lipase immunoreactivity (fPLI) are summarised in (Table 1). Six of the 20 remission cats had International Renal Interest Society (IRIS) stage 2–3 chronic kidney disease (creatinine ≥ 140 μmol/L and USG ≤ 1.035)^2^ at the time of the study.

**Table 1:** Signalment, body condition score, fasting and 2 h glucose concentrations during a GTT for cats in diabetic remission and control cats.

	**Control (*****n*** = **21)**	**Remission (*****n*** = **20)**
**Signalment**		
Sex	Male neutered 12; female speyed 9	Male neutered 12; female speyed 8
Median age at testing (range)	10 years (5–18 years)	13 years (5–19 years)
Median body condition score at testing using 9 point scale (range)	5.5 (4–8)	5.0 (4–7)
Breed	Domestic Short Hair 14, Burmese 4, Oriental 1, Abyssinian 1, Tonkinese 1	Domestic Short Hair 8, Burmese 8, Siamese 2, Australian Mist 1, Russian X 1
Median time in remission at testing (range)	Not applicable	101 days (10 days−4 years)^a^
% with normal fasting glucose concentration (≤ 6.5 mmol/L, 117 mg/dL)	100% (21/21)	80% (16/20)^b^
Mean fasting glucose concentration (range) (mmol/L)	4.4 (2.8–6.1)	5.8 (3.9–8.4)
Mean glucose concentration at 2 h (range) (mmol/L)	8.6 (3.2–16.3)	19.6 (7.6–25.9)^d^
% with normal glucose tolerance test^c^	100% (22/22)	26% (5/19)^d^
Mean fPLI^e^	2.3 (0.5–5.1)^f^	5.8 (1–16)^g^
Mean fructosamine^h^	250 (190–305)^6^	267 (197–347)

a *Time from remission date (14 days after the date insulin administration ceased) to testing date*.

b *The remaining 4 cats (20%) had fasting glucose concentrations of 6.6, 7.5, 7.8, and 8.4 mmol/L (119, 135, 140, and 151 mg/dL)*.

c *Normal glucose tolerance test defined as glucose concentration ≤ 6.5 mmol/L (117 mg /dL) within 3 h after 1 g/kg IV bolus of glucose administered*.

d *The glucose tolerance test was not performed on 1 remission cat*.

e *Laboratory reference range for fPLI was 0.1–3.5 μg/L within the normal range, 3.6–5.3 μg/L may indicate pancreatitis, and >5.4 g/L consistent with pancreatitis*.

f *fPLI and fructosamine results were not available for 1 control cat*.

g *fPLI results were not available for 4 remission cats*.

h *Laboratory reference range for fructosamine was 249–406 μmol/L*.

Three hours after the fasted blood sample was collected and the intravenous catheter placed, cats had a simplified glucose tolerance test performed as has previously described (1, 18). This allowed sufficient time to minimise the effects of stress hyperglycemia following handling (19). In summary, fasting blood glucose was measured using a portable glucometer^3^ calibrated for feline blood, with samples taken from the ear as first preference, or the paw pad or jugular depending on the cat's temperament. A 1 g/kg bolus of glucose (50% glucose wt/vo) was administered intravenously *via* the cephalic catheter. Blood glucose was measured 2 h after glucose administration, and then hourly until blood glucose concentrations had returned to baseline ≤ 6.5 mmol/L (≤ 117 mg/gL), or until 9 h after the start of the test if that occurred first. Food was withheld for the duration of the glucose tolerance test.

Glucose tolerance testing was unable to be performed on one cat that had hormone and metabolomic testing, but it was retained for some analyses in the study. One cats did not have enough sample volume for insulin measurement, two for leptin measurement, and one cat for adiponectin measurement.

### Hormone analysis

Serum leptin concentrations were measured using a commercially available multi-species radioimmunoassay kit^4^, and serum adiponectin and insulin concentrations were measured using ELISA kits^5^,^6^, all of which have previously been validated in cats (20, 21) (see text footnote 2). Intra-assay and inter-assay coefficients of variation (CVs) were <15% and <10% for leptin quality controls of 2.3 and 11.4 ng/mL, respectively. For adiponectin, intra-assay CV was <5% for quality controls of 4.2 and 21.7 μg/mL. Intra and inter-assay reproducibility of feline insulin ELISA kit are <10%.

### Measures of insulin sensitivity

The euglycaemic-clamp and minimal model analysis methods are considered the gold-standard test for determining insulin sensitivity, however this requires an intensive protocol and as enrolled cats were client-owned, it was not feasible in the current study design. Instead standard insulin sensitivity indices were calculated from fasted glucose and insulin concentrations (22). The Homeostasis Model Assessment (HOMA) is the product of glucose and insulin concentrations divided by a constant (23). The higher the HOMA value the lower the insulin sensitivity. The Quantitative Insulin Check Index (QUICKI) is derived from calculating the inverse of the sum of logarithmically expressed values of glucose and insulin (24). The Bennett index is similar to the QUICKI using the inverse function, except with log transformation of the product of insulin and glucose values (25). Lower QUICKI and Bennett indices indicate lower insulin sensitivity.

### Metabolomic analyses

Untargeted metabolomics analyses and metabolite identification *via* Gas Chromatography-Mass Spectrometry (GCMS) and Liquid Chromatography-Mass Spectrometry (LCMS) were performed by Metabolomics Australia (Melbourne, VIC, Australia) as previously described (2).

### Identification of relapse in remission cats

Remission cats were monitored for relapse after testing using casual blood glucose measurements either by home monitoring or during health checks in clinic, with relapse being defined as a blood glucose ≥ 11 mmol/L (198 mg/dL) on a minimum of two occasions, plus clinical signs consistent with diabetes (e.g., polyuria, polydipsia, polyphagia, and weight loss). The endpoint of the study occurred when the cat relapsed, or if the cat did not relapse when they died from other causes, the most recent of the last recorded visit in clinic records (either the referring clinic or the feline-only clinic), or the last contact date with the owner.

### Statistical analyses

Statistical analyses were performed using GraphPad Prism software. Serum hormone concentrations and measures of insulin sensitivity were analysed between diabetic remission and control cats using unpaired *t*-tests. Data were log transformed to remove heterogeneity of variance where required. Spearman's correlation coefficients were obtained to examine the relationship between metabolites previously identified by metabolomics and measures of insulin sensitivity.

## Results

### Glycaemic indicators

All control cats had a fasting blood glucose ≤ 6.5 mmo/L (≤ 117 mg/dL) and normal glucose tolerance [blood glucose ≤ 6.5 mmo/L (≤ 117 mg/dL) at 3 h post glucose administration] (1). The majority (74%, 14/19) of remission cats had impaired glucose tolerance and 20% (4/20) had impaired fasting glucose concentrations (6.6–8.4 mmol/L vs. normal ≤ 6.5 mmol/L; 119–151 mg/dL vs. normal ≤ 117 mg/dL). One remission cat did not reach ≤ 6.5 mmol/L (≤ 117 mg/dL) during the GTT, and had a lowest measured glucose concentration of 7.3 mmol/L (131 mg/dL) at 9 h after glucose administration, which was determined to be its endpoint (1). Six remission cats relapsed within 9 months of follow up. The median time from remission to relapse was 139 days (range, 71–515 days) and the median time from baseline testing to relapse was 81 days (range, 50–257 days) (1).

Four remission cats had a history of corticosteroid administration prior to developing clinical signs and diagnosis of diabetes, one had moderate pancreatitis at diagnosis, and one had severe pancreatitis coupled with corticosteroid administration. Diagnosis of pancreatitis was based on clinical signs and fPLI concentrations measured by an external laboratory. The remaining 14 remission cats were presumed to have type-2 diabetes on diagnosis. Three of the cats with corticosteroid administration without concurrent pancreatitis had impaired glucose tolerance and one had impaired fasting glucose concentrations. Of the two cats with pancreatitis, both (including the one with concurrent corticosteroid administration) had normal fasting glucose and glucose tolerance.

### Hormones and metabolites

Serum insulin and glucose concentrations (Table 2) were significantly increased (*P* < 0.05 and *P* < 0.002, respectively) in remission cats compared to control cats, although the Insulin:Glucose ratio was not significantly different (*P* = 0.44) between the groups. Remission cats exhibited decreased insulin sensitivity, as indicated by a significantly increased HOMA (*P* < 0.01), and significantly decreased QUICKI (*P* < 0.05) and Bennett (*P* < 0.01) indices. However, none of these measures were predictors for diabetic relapse.

**Table 2:** Mean ± SEM (and range) of hormone concentrations and measures of insulin sensitivity for cats in diabetic remission and control cats.

	**Control**	**Remission**	***P*** **-value**
Glucose (mmol/L)	4.5 ± 0.2 (2.8–6.1)	5.9 ± 0.3 (3.9–8.4)	*P* <0.002
Insulin (μU/ml)	15.9 ± 2.1 (3.9–40.2)	25.6 ± 4.2 (4.8–64.2)	*P* <0.05
Insulin to glucose ratio	3.9 ± 0.7	4.7 ± 0.8	N.S.
HOMA	3.07 ± 0.43 (0.71–6.65)	6.77 ± 1.15 (1.17–17.78)	*P* <0.01
QUICKI	0.58 ± 0.0 2 (0.45–0.83)	0.51 ± 0.02 (0.38–0.70)	*P* <0.05
Bennett index	1.53 ± 0.10 (0.89–2.80)	1.13 ± 0.09 (0.65–2.01)	*P* <0.01
Leptin (ng/ml)	19.3 ± 2.8 (1.0–41.0)	32.4 ± 5.4 (6.8–87.7)	*P* <0.05
Adiponectin (μg/ml)	5.6 ± 1.3 (0.2–20.1)	6.5 ± 1.7 (0.1–25.6)	N.S.
Leptin to adiponectin ratio	16.8 ± 5.8	34.0 ± 15.3	N.S.

Adipokine hormone concentrations in remission cats were variable (Table 2), although*-/ leptin concentrations were significantly increased (*P* < 0.05) in remission cats, whilst adiponectin was not significantly different (*P* = 0.68) between remission and control cats. Despite the increase in leptin, the L:A ratio was not significantly different between the groups (*P* = 0.31). Leptin and adiponectin were not predictors for diabetic relapse. Further, there were no significant correlations (absolute *r* values < 0.2, *P* > 0.05) between leptin and either insulin or glucose concentrations, nor any significant relationship between leptin and any measure of insulin sensitivity.

Correlations were examined between hormones, insulin sensitivity indices and the 16 metabolites previously identified by metabolomics (2) as having significant differences between remission and control cats (Table 3). As expected, glucose as measured by GC-MS, had a positive correlation with insulin (*r* = 0.38) and HOMA (*r* = 0.50), and negative correlations with QUICKI (*r* = −0.46) and Bennett (*r* = −0.48). Amino acids that were reduced in remission cats (alanine, asparagine, aspartate, glutamine and valine) had significant positive correlations (0.32 < *r* <0.50) to QUICKI and Bennett, and negative correlations with insulin and HOMA. Glycine, the only amino acid previously found to be increased in remission cats, had significant positive correlations with insulin (*r* = 0.40) and HOMA (*r* = 0.46), and significant negative correlations with QUICKI (*r* = 0.46) and Bennett (*r* = 0.48).

**Table 3:** Correlation analysis (correlation coefficient and *p*-values) between selected metabolites and hormones and measures of insulin sensitivity.

**Metabolite**	**Hormones**				**I to G**	**HOMA**	**QUICKI**	**Bennett**
	**Insulin**	**Leptin**	**Adiponectin**	**L to A**				
GC-MS amino acids								
Alanine	−0.20 NS	−0.21 NS	−0.23 NS	0.09 NS	−0.12 NS	−0.20 NS	0.34 *P* = 0.03	0.36 *P* = 0.03
Asparagine	−0.22 NS	−0.29 NS	−0.09 NS	−0.13 NS	−0.17 NS	−0.24 NS	0.36 *P* = 0.03	0.39 *P* = 0.02
Aspartate	−0.34 *P* = 0.03	−0.42 *P* <0.01	0.10 NS	−0.17 NS	−0.22 NS	−0.34 *P* = 0.03	0.50 *P* <0.01	0.52 *P* <0.001
Glutamine	−0.32 *P* = 0.04	−0.36 *P* = 0.03	0.01 NS	−0.15 NS	−0.17 NS	−0.36 *P* = 0.03	0.44 *P* <0.01	0.48 *P* <0.01
Glycine	0.40 *P* = 0.01	−0.04 NS	−0.17 NS	0.40 *P* = 0.01	0.17 NS	0.46 *P* <0.01	−0.46 *P* <0.01	−0.48 *P* <0.01
Leucine	−0.03 NS	−0.24 NS	−0.10 NS	0.01 NS	0.08 NS	−0.05 NS	0.21 NS	0.25 NS
Methionine	−0.03 NS	−0.19 NS	−0.14 NS	0.01 NS	0.01 NS	0.03 NS	0.09 NS	0.11 NS
Proline	−0.06 NS	−0.13 NS	−0.24 NS	0.07 NS	−0.01 NS	−0.06 NS	0.25 NS	0.29 NS
Serine	0.02 NS	−0.07 NS	−0.24 NS	−0.01 NS	−0.01 NS	0.02 NS	0.14 NS	0.17 NS
Tyrosine	−0.03 NS	−0.15 NS	−0.07 NS	−0.05 NS	0.10 NS	−0.08 NS	0.16 NS	0.23 NS
Valine	−0.16 NS	−0.19 NS	−0.02 NS	−0.03 NS	−0.01 NS	−0.18 NS	0.32 *P* = 0.05	0.36 *P* = 0.02
**GC-MS fatty acids and sterols**								
Stearic acid	−0.19 NS	−0.32 *P* = 0.05	0.02 NS	−0.08 NS	−0.03 NS	−0.28 NS	0.20 NS	0.24 NS
**GC-MS sugars and sugar alcohols**								
Glucose	0.38 *P* = 0.02	0.16 NS	−0.03 NS	0.39 *P* = 0.02	0.32 *P* = 0.05	0.50 *P* <0.01	−0.46 *P* = 0.01	−0.48 *P* <0.01
Xylitol	−0.36 *P* = 0.02	−0.25 NS	0.05 NS	−0.21 NS	−0.19 NS	−0.42 *P* <0.01	0.52 *P* = 0.001	0.57 *P* <0.001
**GC-MS other metabolites**								
Urea	−0.07 NS	0.39 *P* = 0.01	0.25 NS	0.05 NS	−0.10 NS	0.11 NS	−0.04 NS	−0.10 NS
**LC-MS metabolites**								
Carnitine	0.45 *P* <0.01	0.35 *P* = 0.03	−0.10 NS	0.43 *P* <0.01	0.32 *P* = 0.05	0.46 *P* <0.01	−0.47 *P* <0.01	−0.48 *P* <0.01

Another metabolite found to be markedly decreased in remissions cats, the sugar alcohol xylitol, had significant negative correlations with insulin (*r* = −0.36) and HOMA (*r* = −0.42) and positive correlations with QUICKI (*r* = 0.52) and Bennet (*r* = 0.57). In contrast, the fatty acid transporter carnitine, which is substantially increased in remission cats, had positive correlations to insulin (*r* = 0.45) and HOMA (*r* = 0.47), and negative correlations to QUICKI (*r* = −0.48) and Bennett (*r* = −0.49). Urea was not significantly correlated with any measures of insulin resistance (Table 3), even after exclusion of cats with renal disease.

Aspartate, glutamine and stearic acid had significant negative correlations (−0.32 < *r* < −0.42) with leptin, whilst urea and carnitine had significant positive correlations (*r* = 0.39 and 0.35, respectively) with leptin (Table 3). No metabolites exhibited a significant association with adiponectin, although glycine had a significant positive correlation (*r* = 0.40) with the leptin to adiponectin ratio.

## Discussion

The important finding from this study was that cats in diabetic remission are insulin resistant and have increased glucose and insulin concentrations, as well as significant differences in indices of insulin sensitivity. Insulin sensitivity indices were correlated with some metabolites previously identified as being altered in cats in diabetic remission but were not predictive of diabetic relapse.

### Insulin and insulin resistance

Insulin concentrations in cats in diabetic remission were significantly higher than control cats. Remission cats had significant insulin resistance based on HOMA, QUICKI, and Bennett calculations. This indicates that some of the underlying insulin resistance previously demonstrated in diabetic cats still persists once they achieve remission (26). We have previously reported that glucose concentrations are significantly higher in remission cats than control cats, possibly secondary to increased gluconeogenesis (2). While the majority of remission cats (80%) still maintain a normal fasting blood glucose concentration (<6.5 mmol/L; 117 mg/dL), those with fasting blood glucose of ≥7.5 mmol/L are at increased risk of relapse (1).

In humans, HOMA and QUICKI have been shown to have good correlation to the euglycaemic-clamp and minimal model analysis methods (23, 24, 27, 28). In cats, HOMA has been shown to be a good measure of insulin sensitivity (22). A study of 32 cats of varying body weights, including 7 with impaired glucose tolerance, found that fasting insulin concentration and HOMA were useful overall predictors of insulin sensitivity and resistance (22). In obese cats with glucose intolerance, insulin concentrations had the strongest correlations with minimal model analysis, although HOMA, insulin:glucose ratio, and QUICKI were also strongly correlated (22). The Bennett Index was only correlated in overweight and obese cats, and not in mixed weight cats (22). QUICKI has been reported to have a good correlation to both the euglycaemic-clamp and minimal model analysis in lean, obese, and diabetic humans, and had a higher correlation to the euglycaemic-clamp (direct measure of insulin sensitivity) compared to the correlation between the euglycaemic clamp and minimal model (simplified indirect measure) (24).

In normoglycemic humans with normal glucose tolerance, fasting insulin concentration alone has been reported to be as accurate as insulin:glucose ratio, HOMA, and Bennett index in documenting insulin resistance (29). While insulin concentrations are increased initially in insulin resistant states if beta-cell function is normal, eventually failure of pancreatic beta-cells results in insulin concentrations decreasing, while glucose concentrations continue to rise. As a group, diabetic cats in remission have increased glucose concentrations, though the majority maintain normal fasting blood glucose concentration, with 19% of diabetic cats in remission having increased fasting blood glucose concentration (1). However, the majority of these cats have impaired glucose tolerance. This indicates that there was still sufficient pancreatic beta-cell function to maintain normoglycemia in most cats in diabetic remission, in spite of underlying insulin resistance (30).

### Leptin

Leptin concentrations were significantly higher in remission cats. Obese animals and humans have been shown to have increased leptin concentrations in proportion to adipose tissue mass, associated with the development of leptin resistance (11, 14, 31, 32). Decreased serum levels of leptin have been reported with weight loss in cats (32). However, increased leptin concentrations in cats have been associated with insulin resistance independent of obesity (15) and in the current study there was not a significant difference in BCS between remission and control cats. Insulin is a known major regulator of leptin production, and hyperinsulinaemia may result in increased leptin concentrations (15, 33). Remission cats in our study indeed had significantly higher mean insulin concentrations compared to control cats which fits this scenario, although we observed only weak correlations between circulating leptin and insulin, or any measure of insulin sensitivity. Further studies are required to elucidate any potential contribution of insulin insensitivity and hyperinsulinaemia to hyperleptinaemia in diabetic cats.

### Adiponectin

There was no significant difference in adiponectin between remission and control cats, nor was adiponectin significantly correlated with any insulin sensitivity indices. Total adiponectin has been reported as having no correlation to insulin in cats (32). In humans, adiponectin concentrations are reduced in obesity and type 2 diabetes (14). Adiponectin concentrations have been negatively correlated with fasting insulin concentrations and positively correlated with insulin sensitivity, and are considered closely related to the pathophysiology of type 2 diabetes in humans (34, 35). Adiponectin is also decreased in obese and insulin-resistant non-diabetic cats, and decreased further in diabetic cats compared to obese cats (13, 36, 37). Mean BCS between remission and control cats were the same, with no difference in rates of obesity between groups. While cats in remission do have distinct metabolic differences from control cats, they still differ from cats who are overtly diabetic. However, remission cats did have an increase in fasting insulin, which in human type 2 diabetic is associated with a decrease in adiponectin, but this was not demonstrated in remission cats. This study did not differentiate between high and low molecular weight multimers of adiponectin. In humans, the high molecular weight multimer is selectively reduced in the presence of obesity-related states such as type 2 diabetes, and there have been similar findings in cats (9). In cats, high molecular weight adiponectin has been shown to be decreased following feeding of high carbohydrate diets, and the ratio of high to low weight molecular adiponectin is decreased with a higher percentage of body fat (38, 39). Further work in cats is necessary to elucidate the relationship between adiponectin and diabetes, including the role(s) of high molecular weight adiponectin. Certainly total adiponectin concentrations remain unchanged in cats during weight gain or loss (40), and our study found no difference in total adiponectin concentrations between cats in diabetic remission compared to controls when they exhibit similar BCS.

### Metabolomics

We have previously demonstrated that several glucogenic amino acids (i.e., alanine, asparagine, aspartate, glutamine, leucine, methionine, proline, serine, tyrosine, and valine) are decreased in cats in diabetic remission (2). Five of these (i.e., alanine, asparagine, aspartate, glutamine, and valine) had positive correlations with QUICKI and Bennet, and negative correlation with HOMA, indicating that these changes might be associated with insulin resistance in these cats. Glycine was increased in remission cats, and had negative correlations with QUICKI and Bennet and positive correlation with HOMA. This demonstrates a possible link between decreased insulin sensitivity and altered gluconeogenic metabolism in remission cats. Diabetic cats are, on average, six times less sensitive to insulin than normal cats, but hepatic gluconeogenesis is also increased in non-diabetic cats with insulin resistance (3, 41).

Aspartate and glutamine were also negatively correlated with insulin and leptin concentrations, as was stearic acid. It is unknown if an increase in these hormones has a suppressive effect on these metabolites, or that an increase in gluconeogenesis and glucose production depletes these metabolites, leading to increased concentrations of these hormones.

Other metabolites identified with significant correlations were glucose, carnitine, and xylitol. Carnitine was positively correlated to insulin, leptin, L:A, I:G and HOMA, and negatively correlated with QUICKI and Bennet in remission cats. Carnitine concentrations in remission cats were increased even when adjusted for carnitine content of diet (2). Carnitine is synthesised in the liver or absorbed through diet, and supplementation of carnitine in human and rodent diabetic subjects improved glucose parameters (42). Xylitol is reported to be decreased in remission cats, and in our study, had negative correlations with insulin and HOMA and positive correlations with the QUICKI and Bennet (2). Supplementation of 10% xylitol solution fed to diabetic rats reportedly improved glucose tolerance (43, 44). However, while never reported in cats, xylitol ingestion in dogs stimulates insulin secretion resulting in potentially lethal hypoglycaemia (44).

Urea is reported to be increased in remission cats, even when cats with renal disease (IRIS stage 2 and above) were removed from analysis (2). This was hypothesised to be due either to an increase in the glucose-alanine cycle, of which urea is a by-product, or due to underlying renal changes in diabetic cats, which have been demonstrated to have a higher rate of microalbuminaemia compared to non-diabetic cats (70% compared to 18%) (45, 46). Urea was significantly positively correlated with leptin in all cats, and negatively correlated with I:G in cats once those with renal disease were removed. There was no correlation with other measures of insulin sensitivity, indicating that this change in urea may be occurring independently from mechanisms associated with insulin resistance. We have previously reported that urea was associated with impaired glucose tolerance in remission cats and was positively correlated with 2 h glucose concentration and time return to baseline in a GTT (1).

### Limitations

The major limitation in this study was the sample size. A larger group of remission and control cats may have allowed for greater detection of correlations between hormones, insulin resistance, and previously identified metabolites, as well as further possible predictors of relapse. The small sample size also precluded a full analysis of other factors such as age, body condition score, and time in remission to the relationship of these to hormones. The animals included in this study were client owned and were fed a variety of diets, so this study was not able to control for that.

### Conclusions

In conclusion, this study shows that insulin resistance, an underlying factor in diabetic cats, persists in diabetic remission. Leptin was increased despite no significant differences in BCS which is consistent with previous reports in cats that leptin is an indicator of insulin resistance, independent of adiposity as measured by body condition score. Correlations were identified between markers of altered metabolism and insulin resistance in cats in remission, suggesting that insulin resistance and altered gluconeogenic metabolism are linked. While none of these were identified as predictors of relapse, it would be prudent to manage cats in diabetic remission to avoid adding further insulin resistance by normalising body condition and avoiding drugs such as corticosteroids that are known to cause insulin resistance and predispose to diabetes in cats.

## Data Availability Statement

The raw data supporting the conclusions of this article will be made available by the authors, without undue reservation.

## Ethics Statement

The animal study was reviewed and approved by the University of Queensland Animal Ethics Animal Welfare Unit, Research and Research Training Division. Written informed consent was obtained from the owners for the participation of their animals in this study.

## Author contributions

ZR, DD, BB, UR, and KI: revising the article. KI: hormonal analysis. DD, BB, UR, and SG: analysis. DD, BB, UR, SG, SA, and JR: interpretation of data. DD, BB, and UR: metabolomic methodology. JR and SA: research supervision and critically revising the article for important intellectual content. SA: statistical analysis. SG and JR: design. SG: drafting the article for important intellectual content. JR: conception and final approval of the version to be published. All authors contributed to the article and approved the submitted version.

## Funding

The authors declare that this study received funding from Abbot Animal Health and Nestlé Purina. Abbot Animal Health was not involved in the study design, collection, analysis, interpretation of data, the writing of this article, or the decision to submit it for publication.

## Conflict of interest

ZR was employed by the company Nestlé Purina. The remaining authors declare that the research was conducted in the absence of any commercial or financial relationships that could be construed as a potential conflict of interest.

## Publisher's note

All claims expressed in this article are solely those of the authors and do not necessarily represent those of their affiliated organizations, or those of the publisher, the editors and the reviewers. Any product that may be evaluated in this article, or claim that may be made by its manufacturer, is not guaranteed or endorsed by the publisher.

## Footnotes

^1^IDEXX Laboratories, Brisbane, QLD, Australia.

^2^International Renal Interest Society: http://www.iris-kidney.com.

^3^AlphaTRAK, Abbott Animal Health; Cozzi EM et al. (47).

^4^Linco's Multi-Species Leptin Radioimmunoassay (RIA) Kit, Linco Research Inc, St Charles, MO.

^5^B-Bridge International, Otsuka, Tokyo, Japan.

^6^Nippon University.

## References

[1] Gottlieb S Rand J Marshall R Morton J. Glycaemic status and predictors of relapse for diabetic cats in remission. J Vet Intern Med. (2015) 29:184–92. 10.1111/jvim.12509 25418027 PMC4858081

[2] Gottlieb S Rand J Anderson S Morton J Dias D Boughton B. Metabolic profiling of diabetic cats in remission. Front Vet Sci. (2020) 7:218. 10.3389/fvets.2020.00218 32500084 PMC7242727

[3] Feldhahn J Rand J Martin G. Insulin sensitivity in normal and diabetic cats. J Feline Med Surg. (1999) 1:107–15. 10.1016/S1098-612X(99)90067-0 11919024 PMC10822474

[4] Porte D. Beta-cells in type 2 diabetes mellitus. Diabetes. (1991) 40:166–80. 10.2337/diab.40.2.166 1991568

[5] Yang Q Vijayakumar A Kahn B. Metabolites as regulators of insulin sensitivity and metabolism. Nat Rev Mol Cell Biol. (2018) 19:654–72. 10.1038/s41580-018-0044-8 30104701 PMC6380503

[6] Scheen A. Pathophysiology of type 2 diabetes. Acta Clin Belg. (2003) 58:335–40. 10.1179/acb.2003.58.6.001 15068125

[7] Olefsky J. Pathogenesis of insulin resistance and hyperglycaemia in non-insulin dependent diabetes mellitus. Am J Med. (1985) 79 (Suppl. 3B):1–7. 10.1016/S0002-9343(85)80001-2

[8] Barthel A Schmoll D. Novel concepts in insulin regulation of hepatic gluconeogenesis. Am J Physiol Endocrinol Metab. (2003) 285:E685–92. 10.1152/ajpendo.00253.2003 12959935

[9] Lusby A. Characterization of Feline Adiponectin and its Association With Metabolic Indices in Lean and Obese Cats. Knoxville: University of Tennessee (2009).

[10] Coradini M Rand J Morton J Arai T Ishioka K Rawlings J. Fat mass, and not diet, has a large effect on postprandial leptin but not on adiponectin concentrations in cats. Domest Anim Endocrinol. (2013) 45:79–88. 10.1016/j.domaniend.2013.06.001 23827214

[11] Williams M McMillan C Snead E Takada K Chelikani P. Association of circulating adipokine concentrations with indices of adiposity and sex in healthy, adult client owned cats. BMC Vet Res. (2019) 15:332. 10.1186/s12917-019-2080-9 31533709 PMC6749635

[12] Takashima S Nishii N Kato A Matsubara T Shibata H Kitagawa H. Molecular cloning of feline resistin and the expression of resistin, leptin and adiponectin in the adipose tissue of normal and obese cats. J Vet Med Sci. (2016) 78:23–8. 10.1292/jvms.15-0233 26256230 PMC4751112

[13] Zapata R Meachem M Cardosa N Mehain S McMillan C Snead E. Differential circulating concentrations of adipokines, glucagon and adropin in a clinical population of lean, overweight and diabetic cats. BMC Vet Res. (2017) 13:1–9. 10.1186/s12917-017-1011-x 28376869 PMC5379571

[14] Havel P. Control of energy homeostasis and insulin action by adipocyte hormones: leptin, acylation stimulating protein, and adiponectin. Curr Opin Lipidol. (2002) 13:51–9. 10.1097/00041433-200202000-00008 11790963

[15] Appleton D Rand J Sunvold G. Plasma leptin concentrations are independently associated with insulin sensitivity in lean and overweight cats. J Feline Med Surg. (2002) 4:83–93. 10.1053/jfms.2002.0166 12027507 PMC10822654

[16] Ishioka K Omachi A Sasaki N Kimura K Saito M. Feline adiponectin: molecular structures and plasma concentrations in obese cats. J Vet Med Sci. (2009) 71:189–94. 10.1292/jvms.71.189 19262030

[17] Reeve-Johnson M Rand J Vankan D Anderson S Marshall R Morton J. Cutpoints for screening blood glucose concentrations in healthy senior cats. J Feline Med Surg. (2017) 19:1181–91. 10.1177/1098612X16685675 28164734 PMC11104175

[18] Link K Rand J Hendrikz J. Evaluation of a simplified intravenous glucose tolerance test and a reflectance glucose meter for use in cats. Vet Rec. (1997) 140:253–6. 10.1136/vr.140.10.253 9080643

[19] Rand J Kinnaird E Baglioni A Blackshaw J Priest J. Acute stress hyperglycemia in cats is associated with struggling and increased concentrations of lactate and norepinephrine. J Vet Intern Med. (2002) 12:123–32. 10.1111/j.1939-1676.2002.tb02343.x 11899027

[20] Shibata H Sasaki N Honjoh T Ohishi I Takiguchi M Ishioka K. Feline leptin: immunogenic and biological activities of the recombinant protein, and its measurement by ELISA. J Vet Med Sci. (2003) 65:1207–11. 10.1292/jvms.65.1207 14665750

[21] Backus R Havel P Gingerich R Rogers Q. Relationship between serum leptin immunoreactivity and body fat mass as estimated by use of a novel gas-phase Fourier transform infrared spectroscopy deuterium dilution method in cats. Am J Vet Res. (2000) 61:769–801. 10.2460/ajvr.2000.61.796 10895903

[22] Appleton D Rand J Sunvold G. Basal plasma insulin and homeostasis model assessment (HOMA) are indicators of insulin sensitivity in cats. J Feline Med Surg. (2005) 7:183–93. 10.1016/j.jfms.2004.12.002 15922225 PMC10832723

[23] Matthews D Hosker J Rudenski A Naylor B Treacher D Turner R. Homeostasis model assessment: insulin resistance and beta-cell function from fasting plasma glucose and insulin concentrations in man. Diabetologia. (1985) 28:412–9. 10.1007/BF00280883 3899825

[24] Katz A Nambi S Mather K Baron A Sullivan G Quon M. Quantitative insulin sensitivity check index: a simple, accurate method for assessing insulin sensitivity in humans. J Clin Endocrinol Metab. (2000) 85:2402–10. 10.1210/jcem.85.7.6661 10902785

[25] Anderson R Hamman R Savage P Saad M Laws A Kades W. Exploration of simple insulin sensitivity measures derived from frequently sampled intravenous glucose tolerance (FSIGT) tests. The insulin resistance atherosclerosis study. Am J Epidemiol. (1995) 142:724–32. 10.1093/aje/142.7.724 7572943

[26] Scott-Moncrieff J. Insulin resistance in cats. Vet Clin North Am Small Anim Pract. (2010) 40:241–57. 10.1016/j.cvsm.2009.10.007 20219486

[27] Gutch M Kumar S Razi S Gupta K Gupta A. Assessment of insulin sensitivity/resistance. Indian J Endocrinol Metab. (2015) 19:160–4. 10.4103/2230-8210.146874 25593845 PMC4287763

[28] Mather K Hunt A Steinberg H Paradisi G Hook G Katz A. Repeatability characteristics of simple indices of insulin resistance: implications for research applications. J Clin Endocrinol Metab. (2001) 86:5457–64. 10.1210/jcem.86.11.7880 11701722

[29] McAuley K Williams S J M Walker R Lewis-Barned N Temple L. Diagnosing insulin resistance in the general population. Diabetes Care. (2001) 24:460–4. 10.2337/diacare.24.3.460 11289468

[30] Quon M. Editorial: limitations of the fasting glucose to insulin ratio as an index of insulin sensitivity. J Clin Endocrinol Metab. (2001) 86:4617. 10.1210/jcem.86.10.7952 11600512

[31] Appleton D Rand J Sunvold G. Plasma leptin concentrations in cats: reference range, effect on weight gain and relationship with adiposity as measured by dual energy X-ray absorptiometry. J Feline Med Surg. (2000) 2:191–9. 10.1053/jfms.2000.0103 11716618 PMC10829137

[32] Takashima S Nishii N. Concentrations of leptin, adiponectin, and resistin in the serum of obese cats during weight loss. J Vet Med Sci. (2019) 81:1294–300. 10.1292/jvms.19-0091 31366817 PMC6785622

[33] Havel P. Role of adipose tissue in body-weight regulation: mechanisms regulating leptin production and energy balance. Proc Nutr Soc. (2000) 59:359–71. 10.1017/S0029665100000410 10997652

[34] Weyer C Funahashi T Tanaka S Hotta K Matsuzawa Y Pratley R. Hypoadiponectinemia in obesity and type 2 diabetes: closely associated with insulin resistance and hyperinsulinaemia. J Clin Pathol Metab. (2001) 86:1930–5. 10.1210/jcem.86.5.7463 11344187

[35] Kadowaki T Yamauchi T Kubota N Hara K Ueki K Tobe K. Adiponectin and adiponectin receptors in insulin restance, diabetes, and the metabolic syndrome. J Clin Invest. (2006) 116:1784–92. 10.1172/JCI29126 16823476 PMC1483172

[36] Hoenig M Thomaseth K Waldron M Ferguson D. Insulin sensitivity, fat distribution, and adipocytokine response to different diets in lean and obese cats before and after weight loss. Am J Physiol Regul Intergr Comp Med. (2007) 292:R227–34. 10.1152/ajpregu.00313.2006 16902186

[37] Hoenig M Pach N Thomaseth K Le K Schaeffer K Ferguson D. Cats differ from other species in their cytokine and antioxidant enzyme response when developing obesity. Obesity. (2013) 21:E407–14. 10.1002/oby.20306 23408676

[38] Tan H Rand J Morton J Fleeman L Armstrong J Coradini M. Adiponectin profiles are affected by chronic and acute changes in carbohydrate intake in healthy cats. Gen Comp Endocrinol. (2011) 172:468–74. 10.1016/j.ygcen.2011.04.012 21530529

[39] Bjornvad C Rand J Tan H Jensen K Rose F Armstrong J. Obesity and sex influence insulin resistance and total and multimeradiponectin levels in adult neutered domestic shorthair client-owned cats. Domest Anim Endocrinol. (2014) 47:55–64. 10.1016/j.domaniend.2013.11.006 24373250

[40] Witzel A Kirk C Kania S Bartges W Boston R Moyers T. Relationship of adiponectin and its multimers to metabolic indices in cats during weight change. Domest Anim Endocrinol. (2015) 53:70–7. 10.1016/j.domaniend.2015.05.001 26143302

[41] Kley S Hoenig M Glushka J Jin E Burgess S Waldron M. The impact of obesity, sex, and diet on hepatic glucose production in cats. Am J Physiol Regul Integr Comp Physiol. (2009) 269:R936–43. 10.1152/ajpregu.90771.2008 19193946 PMC2698604

[42] Ringseis R Keller J Eder K. Role of carnitine in the regulation of glucose homeostasis and insulin sensitivity: evidence from in vivo and in vitro studies with carnitine supplementation and carnitine deficiency. Eur J Nutr. (2012) 51:1–18. 10.1007/s00394-011-0284-2 22134503

[43] Rahman M Islam M. Xylitol improves pancreatic islets morphology to ameliorate type 2 diabetes in rats: a dose response study. J Food Sci. (2014) 79:H1436. 10.1111/1750-3841.12520 24962431

[44] Peterson M. Xylitol. Top Companion Anim Med. (2013) 28:18–20. 10.1053/j.tcam.2013.03.008 23796483

[45] Layman D Walker D. Potential importance of leucine in treatment of obesity and the metabolic syndrome. J Nutr. (2006) 136:319S−23S. 10.1093/jn/136.1.319S 16365106

[46] Al-Ghazlat S Langston C Greco D Reine N May S Schofer F. The prevalence of microalbuminuria and proteinuria in cats with diabetes mellitus. Top Companion Anim Med. (2011) 26:154–7. 10.1053/j.tcam.2011.04.005 21782146

[47] Cozzi ME Harris CN Lawrence AJ Semon R Shridhara A Matthew MW. Clinical Evaluation of the Hand-Held Abbott Alpha TRAKTM Blood Glucose Monitoring Systems for Use with Dog Cat Blood Samples. (2006). Available online at: www.abbottanimalhealth.com/

